# Editorial: Innovative nutritional technologies for sustainable ruminant farming

**DOI:** 10.3389/fvets.2026.1774700

**Published:** 2026-02-10

**Authors:** Wuyunfeng Zhu, Yitong Liu, Yuxiang Sun, Huiqing Wei, Yuan Lu, Fen Huang, Xin Luo, Kefan Zhang, Xiaoan Zhang, Zijun Zhang, Guanjun Wang, Yafeng Huang

**Affiliations:** 1 College of Animal Science and Technology, Anhui Agricultural University, Hefei, China; 2 Center of Agriculture Technology Cooperation and Promotion of Dingyuan County, Dingyuan, China; 3 Yingshang Agricultural Green Development Promotion Center, Yingshang, China

**Keywords:** functional feed additives, innovative nutrition, methane mitigation, rumen microbiota, sustainable ruminant farming

## Introduction

Ruminant livestock systems play a pivotal role in global food security by providing high-quality animal protein and contributing to agroecosystem stability. However, increasing demand for animal-derived products, coupled with climate change pressures and resource constraints, poses significant challenges to the sustainability of ruminant farming. In this context, nutritional strategies have emerged as one of the most effective and immediately applicable tools to improve production efficiency, enhance animal health, and mitigate environmental impacts. The Research Topic Innovative Nutritional Technologies for Sustainable Ruminant Farming was therefore launched to bring together recent advances that address these challenges through innovative, science-based nutritional interventions.

### Plant-derived nutritional interventions and functional feed additives

Natural plant-derived compounds have attracted increasing interest as sustainable alternatives to conventional feed additives. Within this Research Topic, Zhang et al. demonstrated that supplementation with Euphorbia humifusa extract improved growth performance and modulated serum biomarkers in preweaned calves, highlighting its potential role in early-life nutritional programming and gut health. In addition, Iommelli et al. reported that fennel seed powder supplementation altered milk fatty acid profiles and the expression of genes related to lipid metabolism in dairy goats, supporting the use of plant-based functional feeds to enhance product quality. Collectively, these studies underscore the promise of plant-derived additives in aligning animal productivity with environmentally responsible feeding practices.

### Innovative nutrient delivery technologies and dietary structure optimization

Beyond feed ingredients themselves, the efficiency with which nutrients are delivered and utilized is a critical determinant of sustainable ruminant nutrition. Oliveira et al. provided a comprehensive review of spray-drying microencapsulation as an innovative lipid delivery system, emphasizing its capacity to improve lipid stability, control nutrient release, and enhance metabolic efficiency in ruminant diets. Complementing this technological perspective, Li et al. investigated the effects of dietary neutral detergent fiber (NDF) to non-fiber carbohydrate (NFC) ratios on *in vitro* rumen fermentation, microbial composition, and methane production, demonstrating that optimized dietary structure is a key lever for improving fermentation efficiency while reducing energy losses as methane. Together, these contributions highlight how technological innovation and ration formulation can be integrated to support precision feeding strategies.

### Nutritional regulation of rumen microbiota and host physiological mechanisms

Several contributions in this Research Topic advanced the understanding of how nutritional interventions influence rumen microbial ecosystems and host physiological responses. Yan et al. showed that silage derived from different maize varieties significantly affected growth performance, rumen microbiota composition, and slaughter traits in Hu sheep, reinforcing the importance of forage quality and source in shaping rumen function. Using whole-blood RNA sequencing, Ajiboye et al. revealed immunomodulatory effects of a multi-strain direct-fed microbial in newly weaned beef cattle, providing molecular evidence for the role of probiotics in regulating immune homeostasis. At the cellular level, Bai et al. demonstrated that α-ketoglutaric acid alleviated palmitic acid–induced ferroptosis in sheep endometrial epithelial cells, offering mechanistic insight into how nutritional factors can modulate cellular homeostasis and reproductive health. These studies collectively reflect a shift toward mechanism-driven ruminant nutrition research.

### Environment-adaptive nutrition and methane mitigation

Reducing the environmental footprint of ruminant production, particularly enteric methane emissions, is a major priority for sustainable livestock systems. Wang et al. examined the effects of saline–alkaline grassland degradation on rumen microbial communities and methane emissions, highlighting the strong interactions between environmental conditions, nutritional context, and rumen function. In addition, exploratory studies within this Research Topic, such as the inclusion of unconventional materials (e.g., paper packaging) at low dietary levels, offer novel perspectives on resource recycling and circular feeding systems. Together, these contributions emphasize that environmentally adaptive nutritional strategies are essential for balancing productivity with ecological sustainability.

## Conclusion

Taken together, the articles included in this Research Topic demonstrate that innovative nutritional technologies extend far beyond improving animal performance alone. By integrating plant-derived additives, advanced nutrient delivery systems, dietary structure optimization, and mechanistic insights into host–microbiome interactions, nutrition emerges as a central lever connecting productivity, animal health, and environmental stewardship. These advances align closely with global efforts to achieve sustainable agricultural systems under the framework of climate action and responsible resource use.

